# Assessment of Lipophilicity Parameters of Antimicrobial and Immunosuppressive Compounds

**DOI:** 10.3390/molecules28062820

**Published:** 2023-03-21

**Authors:** Dawid Wardecki, Małgorzata Dołowy, Katarzyna Bober-Majnusz

**Affiliations:** 1 Faculty of Pharmaceutical Sciences in Sosnowiec, Doctoral School, Medical University of Silesia in Katowice, 41-200 Sosnowiec, Poland; 2 Department of Analytical Chemistry, Faculty of Pharmaceutical Sciences in Sosnowiec, Medical University of Silesia in Katowice, 41-200 Sosnowiec, Poland

**Keywords:** antimicrobial drugs, bioactive molecules, chromatography, immunosuppressive compounds, lipophilicity, TLC

## Abstract

Lipophilicity in addition to the solubility, acid-base character and stability is one of the most important physicochemical parameters of a compound required to assess the ADMET properties (absorption, distribution, metabolism, excretion and toxicity) of a bioactive molecule. Therefore, the subject of this work was to determine the lipophilicity parameters of selected antimicrobial and immunosuppressive compounds such as delafloxacin, linezolid, sutezolid, ceftazidime, everolimus and zotarolimus using thin-layer chromatography in reversed phase system (RP-TLC). The chromatographic parameters of lipophilicity (R_MW_) for tested compounds were determined on different stationary phases: RP18F_254_, RP18WF_254_ and RP2F_254_ using ethanol, acetonitrile, and propan-2-ol as organic modifiers of mobile phases used. Chromatographically established R_MW_ values were compared with partition coefficients obtained by different computational methods (AlogPs, AClogP, AlogP, MlogP, XlogP2, XlogP3, logP_KOWWIN_, ACD/logP, milogP). Both cluster and principal component analysis (CA and PCA) of the received results allowed us to compare the lipophilic nature of the studied compounds. The sum of ranking differences analysis (SRD) of all lipophilicity parameters was helpful to select the most effective method of determining the lipophilicity of the investigated compounds. The presented results demonstrate that RP-TLC method may be a good tool in determining the lipophilic properties of studied substances. Obtained lipophilic parameters of the compounds can be valuable in the design of their new derivatives as efficient antimicrobial and immunosuppressive agents.

## 1. Introduction

The search for new therapeutic substances with beneficial pharmacokinetic properties is an important part of the research of many scientists. The systematic research carried out in this direction produces new drugs and their new formulations. In the process of discovery and synthesis of new compounds with potential medical effects, it is important to evaluate their activity. Already in the 19th century it was noted that the activity of a chemical compound is strongly related to its chemical structure. Detailed QSAR (Quantitative Structure-Activity Relationships) studies have been going on continuously since the last century and their goal is to identify and evaluate the relationship between the molecular structure and biological activity of a drug. The method under discussion makes it possible to evaluate the activity of a molecule and also its derivatives. This is an extremely important factor in selecting the most optimal form of a molecule that will have the best effect on the body. The QSAR method also reduces the costs associated with the synthesis process and is therefore economical.

An important step in the search of new drugs as well as new drug formulations is the evaluation of their lipophilicity. Quantitatively, lipophilicity is described by the parameter P, logP or by chromatographic parameter R_MW_ and log k_w_ [1]. Both factors are considered important indicators affecting the biological activity of a molecule. They describe the ability of a drug to pass through biological membranes and to be bound to blood proteins and receptors. An accurate assessment of the lipophilicity of a compound allows us to understand the absorption, distribution, metabolism and elimination (ADME) parameters of the compound. The currently accepted definition of lipophilicity by IUPAC (International Union of Pure and Applied Chemistry) describes the affinity of a molecule for a lipophilic environment.

The classical method for assessing lipophilicity is the shake-flask technique based on splitting a substance into two phases: n-octanol and water [1]. In addition to this method, considered as pioneering, the currently used techniques for assessing lipophilicity are high-performance liquid chromatography (HPLC) and thin-layer chromatography (TLC) in the normal (NP-TLC) and reversed phase system (RP-TLC) [1,2,3,4,5,6]. Chromatographic methods are characterized by rapid execution and high reproducibility of the results obtained, but usually require more expensive equipment (e.g., modern detectors) compared to the shake-flask technique. Among other separation techniques, micellar liquid chromatography (MLC) or micellar electrokinetic chromatography (MEEKC), respectively, in which the mobile phase comprises surfactants that allow better mapping of penetration of molecules through biomembranes can be also be used for lipophilicity determination [7]. The literature review shows that numerous publications have dealt with the subject of determining the lipophilicity of various bioactive compounds using chromatographic methods [8,9,10,11,12,13,14,15,16,17,18,19,20,21,22,23,24,25,26,27,28].

In recent times, intensive development of in silico computational programs for assessing lipophilicity took place. Computational methods are often characterized by good efficiency and reproducibility of experimentally obtained parameters. They are fast and inexpensive, as they do not require laboratory work and reagent consumption compared to experimental methods. They provide a potentially effective tool that can be used for preliminary evaluation of the activity of substances even before chemical synthesis [5].

This work is a continuation of a previously published study [11]. In this paper, the lipophilicity of compounds belonging to two groups of drugs, antimicrobial and immunosuppressive, was analyzed and evaluated. The antimicrobial substances studied were delafloxacin, linezolid, sutezolid and ceftazidime. The immunosuppressive compounds represented were everolimus and zotarolimus. These antimicrobial and immunosuppressive substances are relatively new drugs approved by the U.S. Food and Drug Administration (FDA) in recent years. Therefore, there is no experimental data on the lipophilicity parameters for most of them, e.g., for delafloxacin, sutezolid, everolimus and zotarolimus. Taking into account the importance of the lipophilicity parameter in the design of new drug formulations and its impact on pharmacodynamics and pharmacokinetics profile of the molecule, the present study focuses on determination of this parameter using the TLC method. The experimental results were compared with values of logP obtained from in silico computer databases. In order to compare and to select the best tools for lipophilicity assessment of the studied compounds, chemometric methods such as cluster analysis (CA), principal component analysis (PCA) as well as SRD analysis (sum of ranking differences) were applied.

### Characteristics of Tested Compounds with Antimicrobial and Immunosuppressive Activity

**Figure 1:**
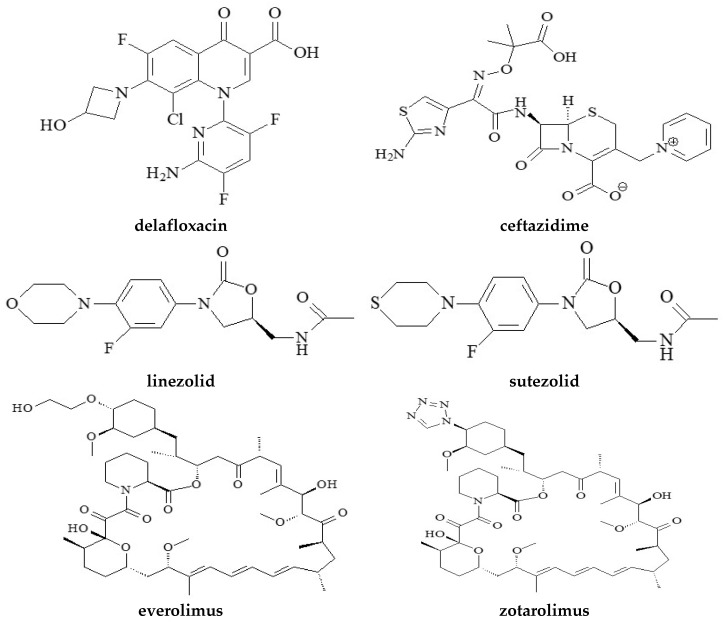
Structures of tested compounds.

The structures of the tested antimicrobial and immunosuppressive drugs are shown in Figure 1. In brief, the tested drug delafloxacin is a relatively new antibiotic belonging to the group of fluoroquinolones. It is a bactericidal substance with a broad spectrum of action. It shows activity against both Gram-positive and Gram-negative bacteria. The FDA approved this compound for medical treatment in 2017 with indications for the treatment of acute bacterial infection of the skin and its structures [29].

The next compound is linezolid. It is a synthetic antibiotic belonging to the oxazolidinone class. This group of compounds was first used in medicine in 1978, and shortly thereafter its antibacterial properties were documented [30]. The drug was officially introduced into the medical field in 1996 [30,31]. It found use in the treatment of hospital-acquired pneumonia caused by *Staphylococcus aureus* and *Streptococcus pneumoniae* and also in the treatment of complicated infections of the skin and its structures [30].

Another studied drug substance is sutezolid. It is an analog of linezolid containing sulfur in the chemical structure (Figure 1). In preclinical in vivo and in vitro studies, it showed promising activity against mycobacteria [31]. Trials performed on mice show an efficacy of this compound comparable to linezolid, so sutezolid could potentially be an effective and novel replacement for the previously used drug linezolid [32].

The last of the four tested antimicrobial compounds is ceftazidime, a third-generation cephalosporin antibiotic administered intravenously. The drug is intended for the treatment of complicated urinary tract infections, including pyelonephritis, as well as pneumonia, and other infections caused mainly by Gram-negative bacteria [33].

The second group of substances tested were immunosuppressant drugs. One of them is everolimus, a potent immunosuppressive drug belonging to the group of oral inhibitors of the mammalian target of rapamycin (mTOR) signal transduction pathway protein kinase. The drug has been approved by the European Medicines Agency (EMA) and FDA for the treatment of hormone-sensitive advanced breast cancer, in the treatment of certain neuroendocrine tumors of pancreatic, gastrointestinal and pulmonary origin, and in the therapy of advanced stage renal cell carcinoma and tuberous sclerosis (TSC) [34,35]. Everolimus has also proven to be an effective and relatively safe adjunctive therapy for controlling epileptic seizures in tuberous sclerosis syndrome [36].

The second immunosuppressive drug tested in this study is zotarolimus, a semisynthetic rapamycin derivative. It is an inhibitor of the mTOR pathway currently indicated as an immunosuppressive drug. It is used in drug-eluting stents to reduce the risk of recurrent vasoconstriction after angioplasty [37,38]. In addition to this, it has potent antiproliferative activity and therefore may be a promising anticancer drug. Studies proved the effectiveness of this drug in treating cancers such as colorectal adenocarcinoma [37].

Table 1 illustrates the selected physicochemical parameters of the studied compounds such as their density, boiling point, index of refraction, molar refractivity, polar surface area, polarizability, surface tension, molar volume, solubility, pK_a_, melting point and storage conditions.

## 2. Results and Discussion

The lipophilicity parameter as a key physicochemical descriptor of the molecule for its pharmacological activity as well as for its transport through biological membranes can be determined both by theoretical means, i.e., calculations, and by experimental methods such as thin-layer chromatography. The two techniques are rapid, simple, and inexpensive ways of performing lipophilicity measurements. Many computer programs can estimate the logP values based on different algorithms. For the six tested compounds belonging to antimicrobial and immunosuppressive agents, the calculated values of logP indices are based on different algorithms: atom-based method (AClogP, XlogP2, XlogP3), fragment contribution (milogP, AlogP), properties dependent methods (AlogPs, MlogP), as well as on the atom based approach and fragmental contribution (logP_KOWWIN_) or, in the case of ACD/logP, on the principle of isolating carbons. Therefore, a critical review of all theoretical logP values and comparison with experimental lipophilicity parameters measured by using the TLC method is useful in selecting the best tool for the lipophilicity assessment of studied compounds. Computational logP values for delafloxacin, linezolid, sutezolid, ceftazidime, everolimus and zotarolimus estimated by using 9 different software are presented in Table 2. The data listed in Table 2 show that the logP value calculated by milogP for delafloxacin and ceftazidime is significantly lower compared to other theoretical logP values and the experimental n-octanol-water partition coefficient (logP_exp_) in the case of ceftazidime. Moreover, the theoretical logP values summarized in Table 2 show that the studied antibiotic substances demonstrate lower lipophilicity compared to immunosuppressive compounds. The lowest value of the lipophilicity parameter is shown by ceftazidime. The available logP_exp_ and calculated logP values are lower than 0 in all cases for this compound except MlogP. The biggest similarity between the theoretical logP values obtained by the 9 software is observed for the two immunosuppressive drugs, i.e., everolimus and zotarolimus. High values of logP for the two drugs in the range from 3.35 (ACD/logP) to 6.87 (AClogP) for everolimus and from 2.75 (MlogP) to 6.50 (AClogP) confirm strong lipophilic properties of both drugs which can have a certain impact on difficulties in their penetration through biological membranes or coronography stent systems.

Analysis of the logP_exp_ value available for linezolid shows the similarity of this descriptor to the theoretical logP values obtained by using most of the software applied in the study, i.e., AlogP, MlogP, XlogP2 and milogP. In the case of ceftazidime the greatest similarity is observed between logP_exp_ and logP_KOWWIN_ as well as AlogPs. Generally, the differences between the theoretical logP values of the studied compounds can be explained by the diversified nature of the algorithms used. Parallel to calculation methods, the chromatographic parameters of lipophilicity of the studied compounds in the form of R_MW_ values were determined. The lipophilicity index measured by the TLC method in a reversed-phase system was calculated based on the R_M_ values determined under different chromatographic conditions, i.e., various stationary and mobile phases, and was next expressed as a value extrapolated to pure water (R_MW_) according to Soczewiński–Wachtmeister’s Equation (1). All chromatographic parameters of the six tested compounds were obtained by using ethanol-water, propan-2-ol-water and acetonitrile-water as mobile phases on chromatographic plates precoated with silica gel RP2F_254_, RP18F_254_ as well as RP18WF_254_ and are presented in Table 3. The statistical results of the Soczewiński–Wachtmeister’s equation are presented in Tables S1–S6 in Supplementary Materials. Analysis of these results confirm the same fact observed during the interpretation of the theoretical data, i.e., calculated logP values, namely that the lipophilic properties of both immunosuppressive drugs, i.e., everolimus and zotarolimus are greater compared to the other drugs. The more hydrophilic properties of ceftazidime are found among the investigated antibiotic agents. The R_MW_ values of everolimus and zotarolimus are placed in the range of 2.2145–3.5637 and 2.1522–3.3623, respectively. Chromatographic parameters of ceftazidime ranged from −2.9545 to 0.9153. The high similarity between the R_MW_ values measured for all tested compounds by using chromatographic plates precoated with silica gel RP18F_254_ as well as RP18WF_254_ should be emphasized.

A certain difference in the R_MW_ values is found using RP2F_254_ plates. These values are relatively lower than those measured by using the same mobile phases on RP18F_254_ as well as RP18WF_254_ plates. This fact can be explained by the influence of chromatographic sorbent activity in form of RP2F_254_ plates. Of all obtained R_MW_ values the best similarity to the n-octanol–water partition coefficient of linezolid (logP_exp_ = 0.9) was achieved on RP2F_254_ developed by using all applied mobile phases consisting of ethanol-water, propan-2-ol and acetonitrile-water. As was highlighted above, in the case of ceftazidime, the retention behavior of this compound depended on the chromatographic conditions used, i.e., chromatographic plate and mobile phase; thus, a wide range of R_MW_ values was obtained. It is, therefore, difficult to indicate the chromatographic parameter which is the most similar to the logP_exp_ value measured in n-octanol-water. The lowest R_MW_ value as in the case of logP_exp_ (−1.6) can be observed for RP18F_254_ plates chromatographed with ethanol-water (R_MW_ = −2.9545). R_MW_ values lower than zero in the range of −0.0357 to −0.6643 could be observed when the RP2F_254_ plates are used (Table 3). These facts confirm the need for optimization of the chromatographic conditions, i.e., selection of appropriate chromatographic plates and mobile phase to obtain reliable results for the lipophilicity descriptor of the studied compounds measured by using the TLC method.

Because of the crucial role of lipophilicity parameters in the prediction of biological activity and drug discovery process, in continuation of this study, the chemometric approach based on cluster analysis (CA) and principal component analysis (PCA) was utilized to compare all theoretical and chromatographic lipophilicity parameters and, next, to group the studied compounds according to their pharmacological activity, i.e., into antibacterial and immunosuppressive classes.

**Figure 2:**
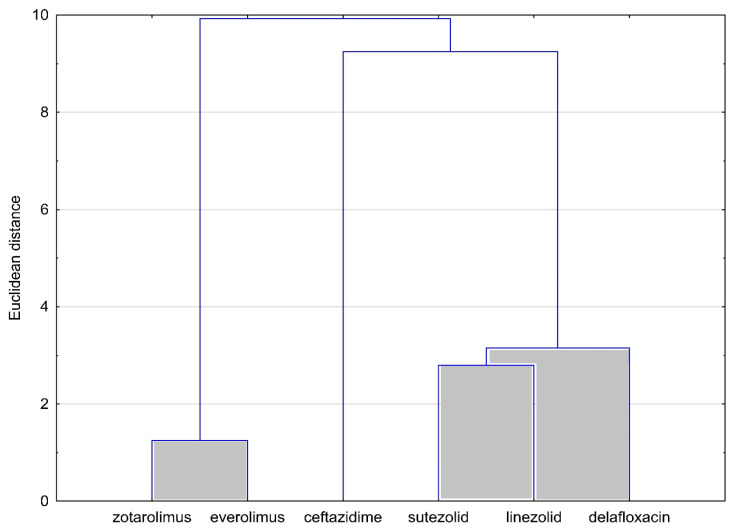
Analysis of the similarities of the tested compounds on the basis of their lipophilicity values.

**Figure 3:**
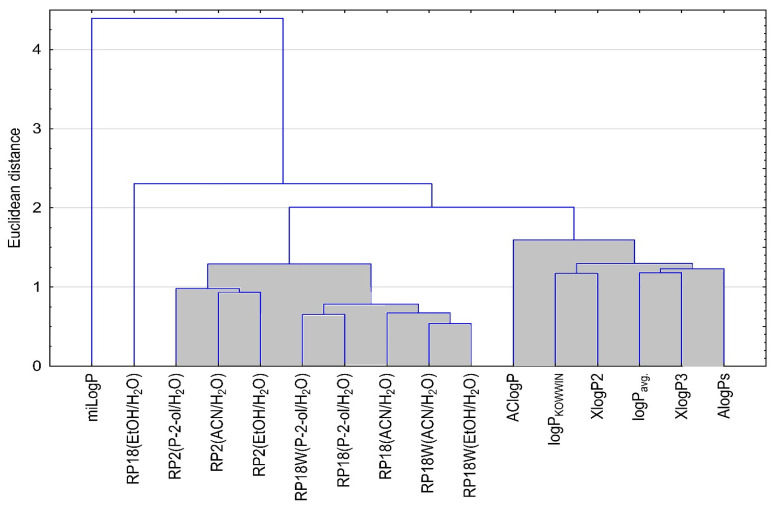
Analysis of similarities of lipophilicity values for tested compounds.

Based on data experimentally obtained by the TLC technique and theoretically calculated using available databases, an analysis of the similarities of the tested compounds was carried out. This analysis was performed twice, analyzing the similarity of the compounds (Figure 2) and analyzing the similarity of the logP values (Figure 3). The results obtained are shown in the following Figure 2 and Figure 3.

The graph of the analysis of the similarities of the compounds in Figure 2 clearly shows two clusters. The first cluster comprises two compounds, namely zotarolimus and everolimus belonging to immunosuppressive agents, and the second cluster comprises three antibacterial compounds: sutezolid, linezolid and delafloxacin. Apart from, of course, similarity in lipophilicity values, the grouped compounds also show another similarity. Two of the first group belong to immunosuppressant substances and have very similar structural structures. On the other hand, three compounds from the second cluster belong to the group of substances with antimicrobial activities and all three have fluorine atoms in their composition. Ceftazidime is the only one of the compounds analyzed to have sulfur atoms in its composition. This explains the fact that it does not belong to any of the clusters identified in the above analysis. Based on the presented facts, it could be concluded that the structural structure affects the lipophilicity values of the compounds tested.

Next, Figure 3 presents the analysis of similarities of lipophilicity values for tested compounds obtained by using different algorithms for all compounds (miLogP, AClogP, logP_KOWWIN_, XlogP2, XlogP3, AlogPs, logP_avg.)_ and employing the TLC method on RP18WF_254_, RP18F_254_ and RP2F_254_ plates developed with the use of ethanol-water (EtOH/H_2_O), propan-2-ol-water (P-2-ol/H_2_O) as well as acetonitrile-water (ACN/H_2_O).

Interestingly, the analysis of similarities in lipophilicity values for the compounds tested shows two main clusters. One includes experimental and the other theoretically calculated lipophilicity parameters values. On this basis, it can be concluded that it is not only the structural structure that affects lipophilicity. The lipophilicity values determined on its basis (theoretical values) differ from those determined in the laboratory. Thus, it can be inferred that the lipophilicity value is also influenced by other factors such as interactions between atoms in the molecule itself and interactions between the compound and the mobile phase used for TLC analysis. The least similar to the others is the theoretical value logP denoted as milogP. This may be affected by how it is calculated based on fragment contributions. These have been obtained by fitting calculated logP with experimental logP for a training set of more than twelve thousand drug-like molecules.

**Figure 4:**
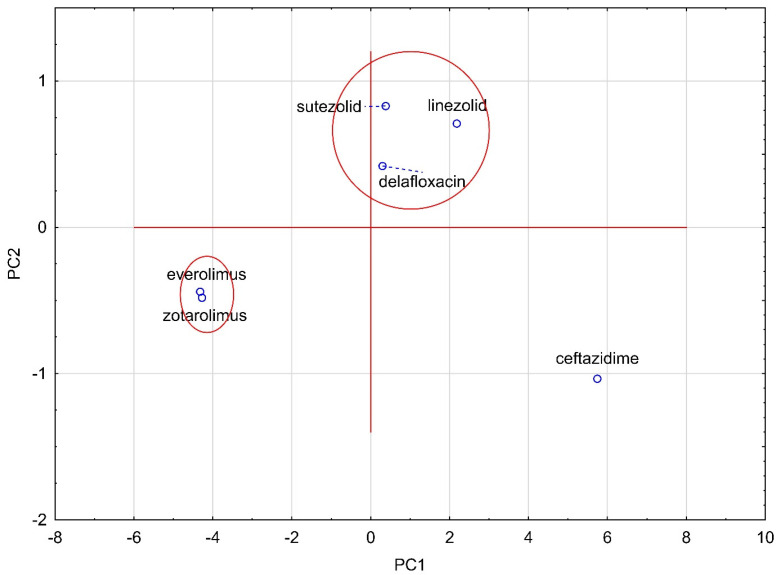
Plot of case projections on the plane of factors for analyzed compounds.

For the obtained lipophilicity values of all analyzed compounds, a principal component analysis (PCA) was also performed. There are 5 main eigenvalues, which describe 100% of the variability of the set of compounds. Determination of the number of main components is based on the scree plot. The Kaiser criterion cannot be applied in this case, since it only points to one main component, which would make it impossible to draw any further conclusions. The following figure shows the plot of case projections on the plane of factors (Figure 4).

The analysis shown in Figure 4 confirms the fact that reducing the number of data to only five leads to the same grouping of compounds as the similarity analysis. The two primary groups include the same compounds as in the similarity analysis, namely zotarolimus and everolimus and sutezolid, linezolid and delafloxacin. Ceftazidime, as the only one containing sulfur atoms in its composition, is on the graph far from the other compounds analyzed.

**Figure 5:**
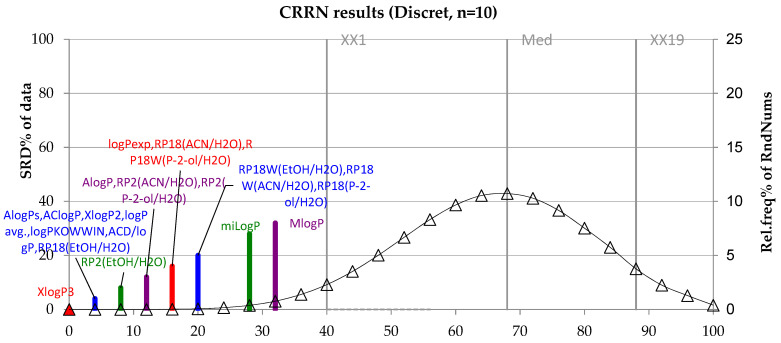
SRD analysis for theoretically and experimentally obtained values of lipophilicity for compounds investigated.

In addition, in order to select the optimal tool for lipophilicity assessment, one of the nonparametric analyses, namely sum of ranking differences (SRD), was performed for the lipophilicity values of the tested compounds. The results presented are shown in the following figure (Figure 5).

Figure 5 presents the sum of ranking differences for all values of lipophilicity for six tested compounds from the groups of immunosuppressant substances (everolimus and zotarolimus) and antimicrobial substances (delafloxacin, ceftazidime, linezolid and sutezolid). Analyzing the graph, it was found that the theoretical value of lipophilicity XlogP3 shows the lowest value. It can therefore be used successfully to determine the lipophilicity of the test group of compounds. Of the theoretical lipophilicity values, MlogP has the highest value and is considered to be the least valuable for determining the lipophilicity of the tested group of compounds. On the other hand, when analyzing the experimentally determined lipophilicity values, the lowest value is shown by the lipophilicity determined using RP18 chromatographic plates and the ethanol-water mobile phase. It is these chromatographic conditions that are most suitable for determining the lipophilicity of the analyzed substances.

## 3. Materials and Methods

### 3.1. Reagents

Ethanol (96%, Reag. Ph Eur.), dimethyl sulfoxide (DMSO), propan-2-ol and acetonitrile of HPLC grades were bought from POCh (Gliwice, Poland). Deionized water was produced using the Direct-Q3 UV system (Millipore, Warsaw, Poland).

### 3.2. Analytes

The reference standards of antimicrobial compounds (purity ≥ 98% HPLC) such as delafloxacin, linezolid, sutezolid and ceftazidime were purchased from Sigma-Aldrich (Beijing, China) and Sigma-Aldrich (St. Louis, MO, USA), respectively. Reference stan-dards of immunosuppressive agents, i.e., everolimus and zotarolimus, were purchased from Sigma-Aldrich (Oakville, Canada). The tested compounds were dissolved in DMSO to a concentration of 5 mg/mL, respectively. Stock solutions were stored at 2–8 °C before analysis.

### 3.3. Chromatographic Materials

Thin-layer chromatography aluminum plates (20 cm × 20 cm, 0.25 mm) precoated with silica gel RP18F_254_, and glass plates coated with silica gel RP18WF_254_ (20 cm × 10 cm) and silica gel RP2F_254_ (10 cm × 10 cm) were obtained from Merck (Darmstadt, Germany).

### 3.4. TLC Analysis

The chromatographic analysis was carried out according to the procedure described earlier [11] on RP-TLC plates (RP2F_254_, RP18F_254_, RP18WF_254_) as a stationary phase using three different mobile phases. Five microliters of standard solutions of analytes were spotted onto the chromatographic plates. The chromatographic chamber 20 cm × 10 cm (Camag, Switzerland) was saturated with the mobile phase vapors for 20 min. The mobile phases were prepared by mixing appropriate organic modifiers (ethanol, propan-2-ol, acetonitrile) and water in different volume compositions. The content of the organic modifier in the mobile phase used was changed in the step of 5% (*v*/*v*) in the range of 30–90% *v*/*v*. Thus, the mobile phases used consisted of a suitable organic solvent and water mixed in the following volume compositions: 30:70; 35:65; 40:60; 45:55; 50:50; 55:55; 60:40; 65:35; 70:30; 75:25; 80:20; 85:15 and 90:10. Chromatograms were developed at room temperature (21 ± 1 °C) to the solvent distance of 7 cm. Next, the chromatograms were dried for 24 h at (21 ± 1 °C) in a fume cupboard. The detection of the studied compounds was carried out under a UV lamp at 254 nm (Camag, Muttenz, Switzerland). The values of R_f_ (retardation factor) are the average values of three independent measurements in each case. To determine the chromatographic parameter of lipophilicity of studied compounds in form of R_MW_, the Soczewiński–Wachtmeister’s [1] equation, which shows the linear relationship between the chromatographic factor R_M_ and volume fraction of organic modifier in the mobile phase (φ) was used [1]:

(1) $$R_{M}={\;R}_{\mathrm{MW}}-b\times \varphi$$

### 3.5. In Silico Calculation of Lipophilicity and Other Physicochemical Parameters

The physicochemical properties of tested compounds such as density, boiling point, index of refraction, molar refractivity, polar surface area, polarizability, surface tension and molar volume given in Table 1 were evaluated by EPIWEB 4.1 program (Estimation Programs Interface) Suite TM Version 4.1 and ChemSpider (http://www.chemspider.com accessed on 2 January 2023). In addition, Table 1 presents other physicochemical parameters of the tested compounds, which besides lipophilicity affect their ADMET properties, such as aqueous solubility, acid-base character (pK_a_ value) as well as stability parameters e.g., melting point and storage conditions in powder and in solution forms that were taken from ChemSpider (http://www.chemspider.com and https://www.selleckchem.com/ accessed on 2 January 2023). Partition coefficients (logP) of six compounds (AlogPs, AlogP, AClogP, MlogP, xlogP2, xlogP3) were calculated according to molecular structures by use of program packages available at Virtual Computational Chemistry Laboratory http://www.vcclab.org./ (accessed on 2 January 2023), while logP_KOWWIN,_ milogP and ACD/logP values were obtained from ChemSpider (http://www.chemspider.com accessed on 2 January 2023). The calculation of logP values for particular molecular structures was based on different methods; atom-based method (AClogP, XlogP2, XlogP3), fragment contribution (milogP, AlogP), properties dependent methods (AlogPs, MlogP), as well as atom-based approach, fragmental contribution (logP_KOWWIN_) and on the principle of isolating carbons such as ACD/logP.

The experimental values of the partition coefficient in n-octanol-water (logP_exp_) available for two of the studied compounds, i.e., for linezolid and ceftazidime, are presented in this work (Table 2). LogP_exp_ values were derived from a drug database online, namely DrugBank (https://www.drugbank.com/ accessed on 2 January 2023). All the experimental and calculated logP values are summarized in Table 2.

### 3.6. Cluster Analysis (CA)

Cluster Analysis, known as clustering, is the method that allows the grouping of objects that are similar to each other [39]. Thanks to a such grouping of objects (data), we find that the data in one cluster, group (cluster) indicate some regularity. Cluster analysis in the presented work was performed using Statistica 13.3 software. During the analysis, the calculations were based on Euclidean distances and the single linkage distance.

### 3.7. Principal Component Analysis (PCA)

The Principal Component Analysis or PCA is chemometric tool useful when a given system is described by many variables. Through PCA, the number of variables can be reduced to the minimum number necessary to describe the variability of the system [40]. The analysis of principal components in the presented work was carried out using the Statistica 13.3 software. The number of eigenvalues was determined based on the Kaiser Criterion and scree plot.

### 3.8. Sum of Ranking Differences Analysis (SRD)

Sum of ranking difference is a useful statistical procedure for comparing methods for the measurements or calculations of the same property, such as lipophilicity descriptors based on ranks [41,42,43]. The SRD analysis of both chromatographically obtained values using various TLC systems (different stationary and mobile phases) and calculated lipophilicity parameter values of examined compounds was performed using Microsoft Excel macro program downloaded at http://aki.ttk.mta.hu/srd/ (accessed on 12 January 2023).

## 4. Conclusions

In this work, the lipophilicity parameters of selected antimicrobial and immunosuppressive compounds, such as delafloxacin, linezolid, sutezolid, ceftazidime, everolimus and zotarolimus have been determined using TLC and calculation methods.

Analyzing these results, it was found that:

- the TLC method can be a good tool for determining the lipophilic properties of investigated substances and their derivatives;
- the obtained lipophilicity parameters (R_MW_ and logP values) indicated that the lowest lipophilic properties were shown by ceftazidime, an antibacterial drug, and the highest by both the tested immunosuppressive drugs; everolimus and zotarolimus;
- chemometric analysis, i.e., CA and PCA, indicated similarities between the tested compounds;
- among all chromatographic parameters, the greatest similarity was observed between the R_MW_ values measured on the RP18F_254_ and RP18WF_254_ plates and, therefore, these plates can be successfully used in lipophilicity studies of the tested drugs interchangeably;
- the results of the SRD analysis of all chromatographic and calculated lipophilicity parameters show that the best tools to evaluate the lipophilicity parameters of the tested compounds are the XlogP3 method and TLC using RP2F_254_ plates and ethanol-water as mobile phase.

It can be suggested that the obtained lipophilicity parameters of these compounds may be valuable in the design of their new derivatives as effective antimicrobial and immunosuppressive agents.

## Figures and Tables

**Table 1:** Physicochemical parameters of tested compounds.

Compound	Density [g/cm^3^]	Solubility in Water [mg/mL]	logS	Boiling Point [°C]	Melting Point [°C]	pK_a_ (Strongest Acidic)	pK_a_ (Strongest Basic)	Index of Refraction	Molar Refractivity [cm^3^]	Polar Surface Area [A°]^2^	Polarizability [cm^3^]	Surface Tension [dyne/cm]	Molar Volume [cm^3^]	Storage in Powder at −20 °C [years]	Storage in Solvent at −80 °C [years]
	Antimicrobial agents														
Delafloxacin	1.8	0.0699	−3.8	698.5	238–241	5.62	−1.3	1.717	96.7	120	38.3	90.5	245.4	3	1
Linezolid	1.3	1.44	−2.4	585.5	176–178	14.85	−1.2	1.554	83.0	71	32.9	47.7	259.0	3	1
Sutezolid	1.3	0.237	−3.2	609.0	229.77	14.85	−0.14	1.584	89.5	87	35.5	50.5	267.5	-	-
Ceftazidime	-	0.00573	−5.0	-	103–113	2.42	4.02	-	-	191	51.0	-	-	3	1
	Immunosuppressive drugs														
Everolimus	1.2	0.00163	−5.8	998.7	-	9.96	−2.7	1.548	257.7	205	102.2	51.4	811.2	3	1
Zotarolimus	1.3	-	-	1016.2	100–105	-	-	1.586	258.1	219	102.3	44.8	769.5	3	1

**Table 2:** Partition coefficients (logP) of studied compounds.

Compound	Atom-Based Methods			Fragment Contribution Methods		Properties Dependent Methods		Atom Based Approach and Fragmental Contribution Methods	Principle of Isolating Carbons Methods	Other logP	
	AC logp	XlogP2	XlogP3	miLogp	AlogP	AlogPs	MlogP	logP_KOWWIN_	ACD/logP	logP_avg_ (Average Value)	logP_exp_ (Experimental Value)
Antimicrobial agents											
Delafloxacin	1.80	3.01	2.67	−0.70	2.66	1.67	2.24	2.26	0.81	2.34	-
Linezolid	0.39	0.89	0.69	0.92	0.89	0.61	0.89	1.26	0.30	0.73	0.9
Sutezolid	0.88	2.05	1.49	1.47	1.64	1.31	1.65	2.12	0.96	1.50	-
Ceftazidime	−0.35	−1.02	−0.21	−5.68	−0.31	−1.21	0.67	−1.34	-	−0.40	−1.60
Immunosuppressive drugs											
Everolimus	6.87	4.09	5.87	4.81	-	5.01	-	4.53	3.35	5.46	-
Zotarolimus	6.50	3.80	5.95	4.55	5.94	4.51	2.75	4.41	3.55	4.91	-

**Table 3:** Comparison of R_MW_ values of tested compounds obtained by using TLC method.

Mobile Phase	Chromatographic Plates		
	RP2F_254_	RP18F_254_	RP18WF_254_
Antimicrobial agents			
Delafloxacin			
Ethanol–water	0.9184	2.1359	1.9694
Propan-2-ol-water	0.7390	1.7719	1.6940
Acetonitrile-water	0.6615	2.3174	1.8351
Linezolid			
Ethanol–water	0.9592	1.1926	1.3407
Propan-2-ol-water	0.8826	0.7884	1.1239
Acetonitrile-water	0.8183	1.5263	1.1927
Sutezolid			
Ethanol–water	1.4334	1.8680	1.9661
Propan-2-ol-water	1.1590	1.5937	1.4784
Acetonitrile-water	1.4312	2.2114	2.0329
Ceftazidime			
Ethanol–water	−0.6643	−2.9545	0.4152
Propan-2-ol-water	−0.3642	0.4691	0.9153
Acetonitrile-water	−0.0357	0.1462	0.4121
Immunosuppressive drugs			
Everolimus			
Ethanol–water	2.9460	3.5637	2.8227
Propan-2-ol-water	2.2145	2.5865	2.5687
Acetonitrile-water	2.8557	2.9462	2.7737
Zotarolimus			
Ethanol–water	3.3623	3.1105	2.7377
Propan-2-ol-water	2.1522	2.4741	2.7821
Acetonitrile-water	2.7480	3.1252	3.2300

## Data Availability

Not applicable.

## Supplementary Materials

The following materials can be downloaded at: www.mdpi.com/article/10.3390/molecules28062820/s1, Table S1: Data for linear correlation (Equation (1)) between R_M_ values and the content of organic modifier in the mobile phase for delafloxacin. Where: correlation coefficient (R^2^), standard error of estimation (SEE); F-factor; significance level (p), volume fraction of organic modifier in mobile phase (φ); Table S2: Data for linear correlation (Equation (1)) between R_M_ values and the content of organic modifier in the mobile phase for linezolid. Where: correlation coefficient (R^2^), standard error of estimation (SEE); F-factor; significance level (p), volume fraction of organic modifier in mobile phase (φ); Table S3: Data for linear correlation (Equation (1)) between R_M_ values and the content of organic modifier in the mobile phase for sutezolid. Where: correlation coefficient (R^2^), standard error of estimation (SEE); F-factor; significance level (p), volume fraction of organic modifier in mobile phase (φ); Table S4: Data for linear correlation (Equation (1)) between R_M_ values and the content of organic modifier in the mobile phase for ceftazidime. Where: correlation coefficient (R^2^), standard error of estimation (SEE); F-factor; significance level (p), volume fraction of organic modifier in mobile phase (φ); Table S5: Data for linear correlation (Equation (1)) between R_M_ values and the content of organic modifier in the mobile phase for everolimus. Where: correlation coefficient (R^2^), standard error of estimation (SEE); F-factor; significance level (p), volume fraction of organic modifier in mobile phase (φ); Table S6: Data for linear correlation (Equation (1)) between R_M_ values and the content of organic modifier in the mobile phase for zotarolimus. Where: correlation coefficient (R^2^), standard error of estimation (SEE); F-factor; significance level (p), volume fraction of organic modifier in mobile phase (φ).
