# Supplementary material for: Assessment of Lipophilicity Parameters of Antimicrobial and Immunosuppressive Compounds
Source: Molecules. 2023 Mar 21;28(6):2820. doi: 10.3390/molecules28062820 (PMC10059999; doi:10.3390/molecules28062820)
Supplement: Supplementary file 1 [file molecules-28-02820-s001.zip › molecules-2277386-supplementary.pdf]

Table S1. Data for linear correlation (Equation 1) between R_M values and the content of organic modifier in the mobile phase for delafloxacin. Where: correlation coefficient (R^2), standard error of estimation (SEE); F-factor; significance level (p), volume fraction of organic modifier in mobile phase (φ).

Stationary phase	R_{MW}±SE	b±SE	R^2	SEE	F	p	φ
**Ethanol – water (v/v)**							
$RP18F_{254}$	2.1359±0.2132	3.1130±0.3492	90.85	0.1929	79.48	0.0000	0.30 – 0.90
$RP18WF_{254}$	1.9694±0.2233	2.9218±0.3658	88.86	0.2021	63.80	0.0000	0.30 – 0.90
$RP2F_{254}$	0.9184±0.2056	1.9995±0.3368	81.50	0.1861	35.25	0.0003	0.30 – 0.90
**Acetonitrile – water (v/v)**							
**Stationary phase**	**R_{MW}±SE**	**b±SE**	**R^2**	**SEE**	**F**	**p**	**φ**
$RP18F_{254}$	2.3174±0.2273	3.7385±0.4261	92.77	0.1505	76.98	0.0001	0.30 – 0.70
$RP18WF_{254}$	1.8351±0.1579	3.0359±0.3124	94.97	0.0922	94.44	0.0002	0.30 – 0.65
$RP2F_{254}$	0.6615±0.0838	1.6300±0.1758	95.55	0.0424	86.00	0.0008	0.30 – 0.60
**Propan-2-ol – water (v/v)**							
**Stationary phase**	**R_{MW}±SE**	**b±SE**	**R^2**	**SEE**	**F**	**p**	**φ**
$RP18F_{254}$	1.7719±0.1543	3.3049±0.2892	95.61	0.1021	130.55	0.0000	0.30 – 0.70
$RP18WF_{254}$	1.6940±0.1578	3.2557±0.2584	95.20	0.1428	158.70	0.0000	0.30 – 0.90
$RP2F_{254}$	0.7390±0.0916	2.0590±0.1609	95.90	0.0711	163.67	0.0000	0.30 – 0.80

Table S2. Data for linear correlation (Equation 1) between R_M values and the content of organic modifier in the mobile phase for linezolid. Where: correlation coefficient (R^2), standard error of estimation (SEE); F-factor; significance level (p), volume fraction of organic modifier in mobile phase (φ).

Stationary phase	R_{MW}±SE	b±SE	R^2	SEE	F	p	φ
**Ethanol – water (v/v)**							
$RP18F_{254}$	1.1926±0.1197	2.1316±0.1961	93.66	0.1083	118.19	0.0000	0.30 – 0.90
$RP18WF_{254}$	1.3407±0.1844	2.5770±0.3020	90.10	0.1668	72.82	0.0000	0.30 – 0.90
$RP2F_{254}$	0.9592±0.1053	2.1849±0.1724	95.25	0.0953	160.55	0.0000	0.30 – 0.90
**Acetonitrile – water (v/v)**							
**Stationary phase**	**R_{MW}±SE**	**b±SE**	**R^2**	**SEE**	**F**	**p**	**φ**
$RP18F_{254}$	1.5263±0.1071	2.7846±0.2246	97.46	0.0542	153.67	0.0002	0.30 – 0.60
$RP18WF_{254}$	1.1927±0.1187	2.0267±0.2225	93.26	0.0785	83.00	0.0001	0.30 – 0.70
$RP2F_{254}$	0.8183±0.0766	1.6660±0.1605	96.42	0.0388	107.70	0.0005	0.30 – 0.60
**Propan-2-ol – water (v/v)**							
**Stationary phase**	**R_{MW}±SE**	**b±SE**	**R^2**	**SEE**	**F**	**p**	**φ**
$RP18F_{254}$	0.7884±0.0661	1.7600±0.1161	97.04	0.0513	229.72	0.0000	0.30 – 0.80
$RP18WF_{254}$	1.1239±0.1911	2.4862±0.3131	88.74	0.1730	63.05	0.0000	0.30 – 0.90
$RP2F_{254}$	0.8826±0.0794	2.2085±0.1301	97.30	0.0719	288.20	0.0000	0.30 – 0.90

Table S3. Data for linear correlation (Equation 1) between R_M values and the content of organic modifier in the mobile phase for sutezolid. Where: correlation coefficient (R^2), standard error of estimation (SEE); F-factor; significance level (p), volume fraction of organic modifier in mobile phase (φ).

Stationary phase	R_{MW}±SE	b±SE	R^2	SEE	F	p	φ
**Ethanol – water (v/v)**							
$RP18F_{254}$	1.8680±0.1436	2.7763±0.2352	94.57	0.1299	139.38	0.0000	0.30 – 0.90
$RP18WF_{254}$	1.9661±0.2003	3.2236±0.3280	92.35	0.1812	96.57	0.0000	0.30 – 0.90
$RP2F_{254}$	1.4334±0.1648	2.6154±0.2699	92.15	0.1491	93.92	0.0000	0.30 – 0.90
**Acetonitrile – water (v/v)**							
**Stationary phase**	**R_{MW}±SE**	**b±SE**	**R^2**	**SEE**	**F**	**p**	**φ**
$RP18F_{254}$	2.2114±0.1711	3.6252±0.3387	95.82	0.1000	114.57	0.0001	0.30 – 0.65
$RP18WF_{254}$	2.0329±0.1073	3.2897±0.2123	97.96	0.0627	240.20	0.0000	0.30 – 0.65
$RP2F_{254}$	1.4312±0.2173	2.6897±0.4556	89.71	0.1100	34.86	0.0041	0.30 – 0.60
**Propan-2-ol – water (v/v)**							
**Stationary phase**	**R_{MW}±SE**	**b±SE**	**R^2**	**SEE**	**F**	**p**	**φ**
$RP18F_{254}$	1.5937±0.0834	2.8753±0.1562	98.26	0.0552	338.61	0.0000	0.30 – 0.70
$RP18WF_{254}$	1.4784±0.0282	2.7391±0.0528	99.78	0.0187	2687.10	0.0000	0.30 – 0.70
$RP2F_{254}$	1.1590±0.0997	2.4285±0.1633	96.51	0.0902	221.18	0.0000	0.30 – 0.90

Table S4. Data for linear correlation (Equation 1) between R_M values and the content of organic modifier in the mobile phase for ceftazidime. Where: correlation coefficient (R^2), standard error of estimation (SEE); F-factor; significance level (p), volume fraction of organic modifier in mobile phase (φ).

Stationary phase	R_{MW}±SE	b±SE	R^2	SEE	F	p	φ
**Ethanol – water (v/v)**							
$RP18F_{254}$	-2.9545±0.2885	-3.3446±0.5317	95.19	0.0786	39.57	0.0244	0.40 – 0.65
$RP18WF_{254}$	0.4152±0.1205	1.0246±0.2117	76.99	0.0935	23.42	0.0019	0.30 – 0.80
$RP2F_{254}$	-0.6643±0.0179	1.9533±0.0385	99.96	0.0082	2575.47	0.0125	0.30 – 0.60
**Acetonitrile – water (v/v)**							
**Stationary phase**	**R_{MW}±SE**	**b±SE**	**R^2**	**SEE**	**F**	**p**	**φ**
$RP18F_{254}$	0.1462±0.1058	1.4131±0.2525	94.00	0.0373	31.33	0.0305	0.30 – 0.50
$RP18WF_{254}$	0.4121±0.1287	1.3463±0.2213	90.24	0.0463	36.99	0.0037	0.45 – 0.70
$RP2F_{254}$	-0.0357±0.2291	3.0260±0.4804	90.84	0.1160	39.67	0.0032	0.30 – 0.60
**Propan-2-ol – water (v/v)**							
**Stationary phase**	**R_{MW}±SE**	**b±SE**	**R^2**	**SEE**	**F**	**p**	**φ**
$RP18F_{254}$	0.4691±0.3767	3.1340±0.6225	92.69	0.0984	25.35	0.0373	0.50 – 0.70
$RP18WF_{254}$	0.9153±0.0236	3.9500±0.0577	99.98	0.0082	4680.75	0.0093	0.30 – 0.60
$RP2F_{254}$	-0.3642±0.0639	1.9300±0.0981	99.74	0.0069	386.67	0.0323	0.60 – 0.80

Table S5. Data for linear correlation (Equation 1) between R_M values and the content of organic modifier in the mobile phase for everolimus. Where: correlation coefficient (R^2), standard error of estimation (SEE); F-factor; significance level (p), volume fraction of organic modifier in mobile phase (φ).

Stationary phase	R_{MW}±SE	b±SE	R^2	SEE	F	p	φ
**Ethanol – water (v/v)**							
$RP18F_{254}$	3.5637±0.4934	4.4741±0.6948	91.20	0.2026	41.46	0.0030	0.55 – 0.90
$RP18WF_{254}$	2.8227±0.2705	3.7623±0.3955	94.76	0.1366	90.47	0.0002	0.50 – 0.90
$RP2F_{254}$	2.9460±0.2792	4.1739±0.4082	95.44	0.1410	104.55	0.0002	0.50 – 0.90
**Acetonitrile – water (v/v)**							
**Stationary phase**	**R_{MW}±SE**	**b±SE**	**R^2**	**SEE**	**F**	**p**	**φ**
$RP18F_{254}$	2.9462±0.1616	3.3324±0.2362	97.55	0.0816	199.04	0.0000	0.50 – 0.90
$RP18WF_{254}$	2.7737±0.0865	3.7501±0.1401	99.31	0.0414	716.43	0.0000	0.45 – 0.80
$RP2F_{254}$	2.8557±0.3023	4.1954±0.5201	94.21	0.1088	65.07	0.0013	0.45 – 0.70
**Propan-2-ol – water (v/v)**							
**Stationary phase**	**R_{MW}±SE**	**b±SE**	**R^2**	**SEE**	**F**	**p**	**φ**
$RP18F_{254}$	2.5865±0.3308	4.0314±0.5565	89.74	0.1965	52.48	0.0004	0.40 – 0.80
$RP18WF_{254}$	2.5687±0.1728	4.2893±0.3091	97.47	0.0818	192.61	0.0000	0.40 – 0.70
$RP2F_{254}$	2.2145±0.2158	3.8021±0.3631	94.81	0.1282	109.66	0.0000	0.40 – 0.80

Table S6. Data for linear correlation (Equation 1) between R_M values and the content of organic modifier in the mobile phase for zotarolimus. Where: correlation coefficient (R^2), standard error of estimation (SEE); F-factor; significance level (p), volume fraction of organic modifier in mobile phase (φ).

Stationary phase	R_{MW}±SE	b±SE	R^2	SEE	F	p	φ
**Ethanol – water (v/v)**							
$RP18F_{254}$	3.1105±0.4265	4.0724±0.6007	91.99	0.1751	45.96	0.0025	0.55 – 0.90
$RP18WF_{254}$	2.7377±0.3661	3.7065±0.5156	92.82	0.1503	51.68	0.0020	0.55 – 0.90
$RP2F_{254}$	3.3623±0.2125	4.7845±0.3224	97.35	0.1298	220.26	0.0000	0.45 – 0.90
**Acetonitrile – water (v/v)**							
**Stationary phase**	**R_{MW}±SE**	**b±SE**	**R^2**	**SEE**	**F**	**p**	**φ**
$RP18F_{254}$	3.1252±0.0939	3.5524±0.1373	99.26	0.0474	669.48	0.0000	0.50 – 0.90
$RP18WF_{254}$	3.2300±0.2319	4.4507±0.3757	96.56	0.1109	140.31	0.0001	0.45 – 0.80
$RP2F_{254}$	2.7480±0.3069	3.6464±0.4971	91.50	0.1467	53.80	0.0007	0.45 – 0.80
**Propan-2-ol – water (v/v)**							
**Stationary phase**	**R_{MW}±SE**	**b±SE**	**R^2**	**SEE**	**F**	**p**	**φ**
$RP18F_{254}$	2.4741±0.1837	4.0474±0.3160	97.62	0.0661	164.04	0.0002	0.45 – 0.70
$RP18WF_{254}$	2.7821±0.1585	4.7646±0.2980	98.46	0.0623	255.70	0.0001	0.40 – 0.65
$RP2F_{254}$	2.1522±0.1723	3.7045±0.2711	96.39	0.1257	186.77	0.0000	0.40 – 0.90

Supplementary Materials: The following materials can be downloaded at: www.mdpi.com/xxx/s1, Table S1: Data for linear correlation (Equation 1) between R_M values and the content of organic modifier in the mobile phase for delafloxacine. Where: correlation coefficient (R^2), standard error of estimation (SEE); F-factor; significance level (p), volume fraction of organic modifier in mobile phase (φ); Table S2: Data for linear correlation (Equation 1) between R_M values and the content of organic modifier in the mobile phase for linezolid. Where: correlation coefficient (R^2), standard error of estimation (SEE); F-factor; significance level (p), volume fraction of organic modifier in mobile phase (φ); Table S3: Data for linear correlation (Equation 1) between R_M values and the content of organic modifier in the mobile phase for sutezolid. Where: correlation coefficient (R^2), standard error of estimation (SEE); F-factor; significance level (p), volume fraction of organic modifier in mobile phase (φ); Table S4: Data for linear correlation (Equation 1) between R_M values and the content of organic modifier in the mobile phase for ceftazidime. Where: correlation coefficient (R^2), standard error of estimation (SEE); F-factor; significance level (p), volume fraction of organic modifier in mobile phase (φ); Table S5: Data for linear correlation (Equation 1) between R_M values and the content of organic modifier in the mobile phase for everolimus. Where: correlation coefficient (R^2), standard error of estimation (SEE); F-factor; significance level (p), volume fraction of organic modifier in mobile phase (φ); Table S6: Data for linear correlation (Equation 1) between R_M values and the content of organic modifier in the mobile phase for zotarolimus. Where: correlation coefficient (R^2), standard error of estimation (SEE); F-factor; significance level (p), volume fraction of organic modifier in mobile phase (φ).
